# A Resected Case of Metachronous Gallbladder Metastasis of Gastric Cancer Mimicking Gallbladder Cancer

**DOI:** 10.70352/scrj.cr.25-0093

**Published:** 2025-06-24

**Authors:** Ryohei Matsumoto, Koichiro Haruki, Masami Yuda, Yoshihiro Shirai, Masashi Tsunematsu, Shinji Onda, Michinori Matsumoto, Kenei Furukawa, Fumiaki Yano, Toru Ikegami

**Affiliations:** 1 Division of Hepatobiliary and Pancreatic Surgery, Department of Surgery, The Jikei University School of Medicine, Tokyo, Japan; 2 Division of Gastrointestinal Surgery, Department of Surgery, The Jikei University School of Medicine, Tokyo, Japan

**Keywords:** gastric cancer, gallbladder metastasis, gallbladder cancer

## Abstract

**INTRODUCTION:**

Gallbladder metastases from malignancies, including gastric cancer, are extremely rare. Diagnosis is challenging due to the lack of specific symptoms and the absence of distinctive radiological features that can differentiate metastatic lesions from primary gallbladder tumors.

**CASE PRESENTATION:**

An 81-year-old male was diagnosed as early gastric cancer and underwent endoscopic submucosal dissection and additional laparoscopic proximal gastrectomy for residual tumor and lymph node metastasis 5 years prior. Following adjuvant chemotherapy, the patient underwent multiple interventions for metastatic disease, including liver resection for liver metastasis of segment 2/3, radiofrequency ablation for liver metastasis of segment 5/6, and lobectomy of the right middle lobe for lung metastasis. During follow-up, a nodular lesion was newly detected at the gallbladder fundus through computed tomography. Diagnostic imaging, including endoscopic ultrasonography and Gd-EOB-DTPA, suggested a potential gallbladder cancer with sub-serosal lesion. We performed an extended cholecystectomy lymph node dissection, and pathological examination revealed the tumor to be a gallbladder metastasis from the original gastric cancer, confirmed through immunohistochemical staining.

**CONCLUSIONS:**

We herein report a rare case of metachronous gallbladder metastasis from gastric cancer. Preoperative diagnosis of gallbladder metastasis is challenging due to its radiological similarity to primary gallbladder cancer.

## Abbreviations

CT
: computed tomography
ESD
: endoscopic submucosal dissection
EUS
: endoscopic ultrasonography
MRI
: magnetic resonance imaging

## INTRODUCTION

Gallbladder metastases from malignancies, including gastric cancer, are extremely rare.^1)^ The incidence of gallbladder metastasis from gastric cancer has been reported to be 0.06%.^2)^ Since it does not present with specific symptoms until the development of acute cholecystitis due to obstruction of the cystic duct, the diagnosis is often delayed and may be discovered at autopsy.^3,4)^ In addition, diagnosis of gallbladder metastasis is often difficult due to the lack of specific radiological findings to distinguish from other gallbladder diseases, including gallbladder cancer.^3,5,6)^ We herein report a resected case of metachronous gallbladder metastasis of gastric cancer mimicking gallbladder cancer.

## CASE PRESENTATION

**Fig. 1:**
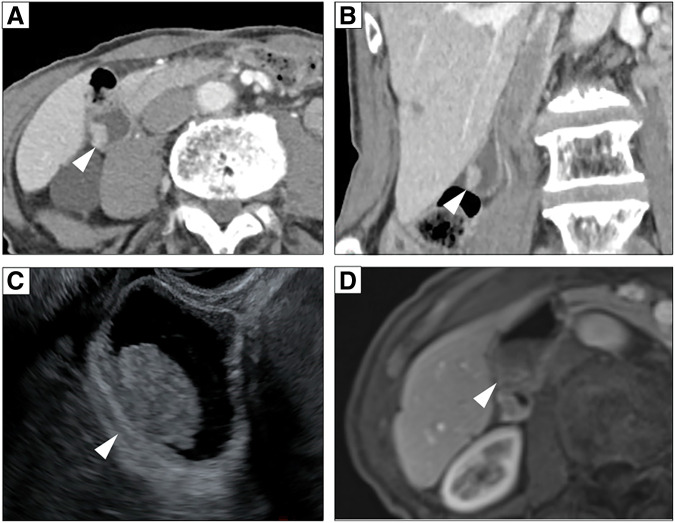
Preoperative imaging. Enhanced computed tomography and T2-weighted magnetic resonance imaging and endoscopic ultrasonography. (**A**, **B**) Contrast-enhanced computed tomography showed a nodular lesion at the fundus of the gallbladder (arrowheads). (**C**) Endoscopic ultrasound showed a protruding lesion of 22 mm at the fundus of the gallbladder and which had sub-serosal invasion (arrowhead). (**D**) Gd-EOB-DTPA enhanced magnetic resonance showed a nodular tumor at the fundus of the gallbladder without evidence of lymph node or distant metastasis (arrowhead).

**Fig. 2:**
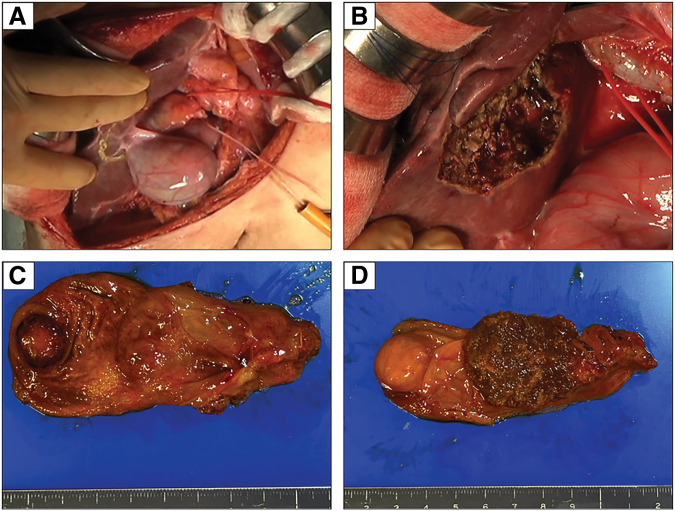
Operative findings and resected specimen. (**A**) A tumor was detected at the fundus of the gallbladder, and there were no adhesions or inflammation around the gallbladder. (**B**) After extended cholecystectomy. (**C**, **D**) The resection specimen showed a 15 × 12 mm pedunculated tumor at the fundus of the gallbladder.

**Fig. 3:**
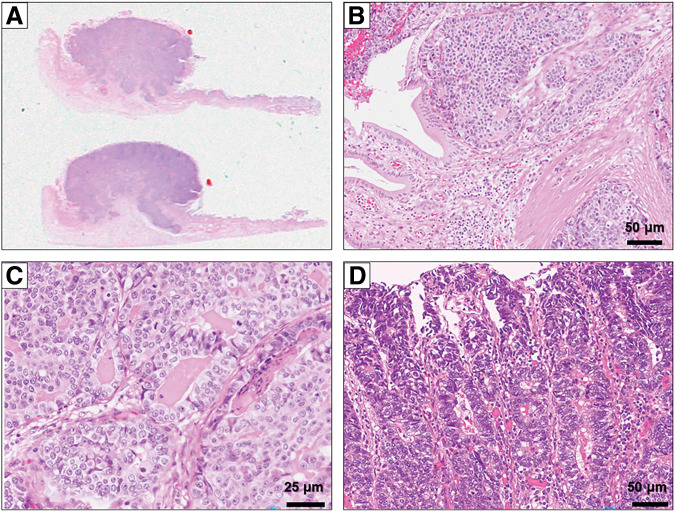
Pathological findings (HE). (**A**) A pedunculated tumor measuring 15 × 12 mm at the gallbladder fundus (macroscopic view). (**B**) Atypical cells proliferate in a tubular and nested pattern, predominantly involving the epithelium and stroma. Non-neoplastic epithelium remained near the base. The tumor shows invasive growth from the subepithelial region to the subserosal layer (×200). (**C**) The tumor cells had a glandular epithelial appearance (×400). (**D**) The histological features of the gallbladder lesion were comparable to the patient’s previously diagnosed gastric adenocarcinoma (×200). HE, hematoxylin-eosin staining

**Fig. 4:**
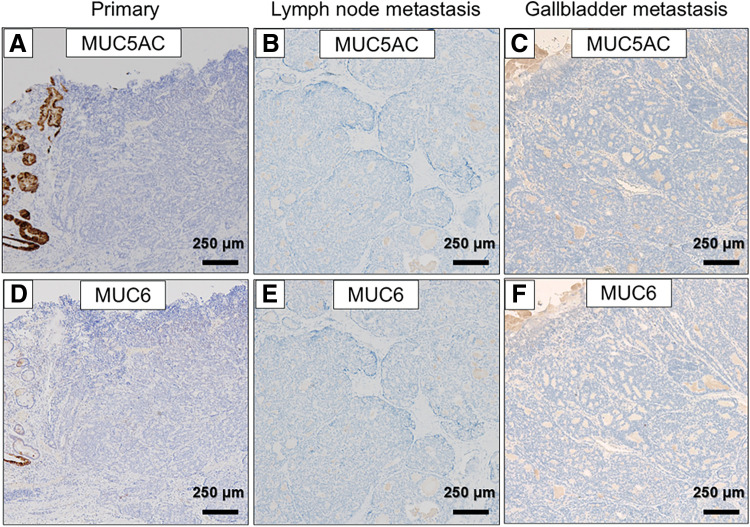
Immunohistochemical staining of MUC5AC and MUC6. MUC5AC (**A**–**C**) and MUC6 (**D**–**F**) were negative in both the primary gastric cancer, the lymph node metastases, and the gallbladder metastasis (×40).

**Fig. 5:**
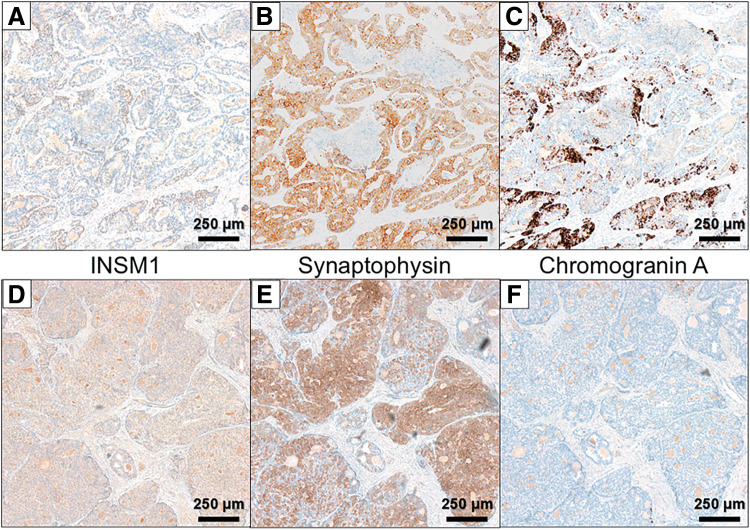
Immunohistochemical staining of INSM1, chromogranin A, and synaptophysin. The tumor showed partial positivity for INSM1 and chromogranin A, and positive staining for synaptophysin in both lymph node metastases (**A**–**C**) and the gallbladder metastasis (**D**–**F**) (×40).

An 81-year-old male was diagnosed as early gastric cancer and underwent ESD 5 years ago. Due to the invasion to submucosa of more than 2250 μm [por, T1b, ly3, v2, UL0, pHM (3.5 mm), pVM0 (0.2 mm)], he was referred to our hospital for radical gastrectomy. He then underwent laparoscopic proximal gastrectomy 4 months after ESD, and the pathological examination revealed that there were residual cancer cells in the scar with lymph node metastases, and the final disease stage was T2N1M0, Stage IIA [tub1, ly0, v0, pPM0 (3 mm), pDM0 (8 mm), with metastases of left and right paracardial lymph node]. Adjuvant chemotherapy with XELOX regimen was performed. However, during adjuvant chemotherapy, a solitary liver metastasis was found in segment 2/3 of the liver 4 years ago, and the laparoscopic left lateral sectionectomy was performed. He continued to receive chemotherapy, however, due to grade 3 hand-foot syndrome induced by capecitabine, the regimen was switched to SOX regimen. Three years ago, he developed a solitary liver metastasis in the segment of 5/6, for which the radiofrequency ablation was performed. After that, he was followed up without chemotherapy, however, a solitary lung metastasis was detected in segment 4 of the right lung 1 year ago. Robotic-assisted thoracoscopic lobectomy of the right middle lobe was performed. In this time, a nodular lesion at the fundus of the gallbladder was newly found by a CT during follow-up. The lesion showed wall thickening and delayed enhancement at the fundus of the gallbladder, suggesting a gallbladder tumor (**Fig. 1A**, **1B**). It protruded into the lumen, consistent with gallbladder cancer. A blood test showed that the inflammatory marker was not elevated, serum carcinoembryonic antigen level was 4.4 ng/mL, and serum carbohydrate antigen 19-9 level was 35 U/mL. EUS revealed a protruding lesion of 22 mm at the fundus of the gallbladder, suspicious for flat infiltrating-type gallbladder cancer. The outer hyperechoic layer was irregular, indicating subserosal invasion (**Fig. 1C**). Gd-EOB-DTPA enhanced MRI imaging showed a nodular tumor at the fundus of the gallbladder without evidence of lymph node or distant metastasis (**Fig. 1D**). According to these findings, we diagnosed the tumor as gallbladder cancer with subserosal invasion (cT2N0M0, Stage IIA), and we decided to perform extended cholecystectomy. After laparotomy, there was no peritoneal metastasis or liver metastasis. A tumor was detected at the fundus of the gallbladder, and there were no adhesions or inflammation around the gallbladder. We performed cholecystectomy with wedge resection of the gallbladder bed and lymph node dissection of the hepatoduodenal ligament (**Fig. 2A**, **2B**). The operative time was 86 min, and the intraoperative blood loss was 10 mL. The resection specimen showed a 15 × 12 mm pedunculated tumor at the fundus of the gallbladder (**Fig. 2C**, **2D**). Pathological examination revealed adenocarcinoma with invasion from the submucosal to the subserosal layers. Tumor cells proliferated in a tubular and cystic pattern mainly from the subepithelial region. The non-neoplastic epithelium remained at the base of the tumor, suggesting the tumor did not originate from the gallbladder mucosa. The tumor cells were relatively tall and glandular, and morphologically resembled the patient’s primary gastric tumor (**Fig. 3A**–**3D**). Immunohistochemistry revealed that MUC5AC and MUC6 were negative in primary gastric cancer, lymph node metastases, and gallbladder lesions (**Fig. 4A**–**4F**). As partial neuroendocrine differentiation was suspected, staining for INSM1, chromogranin A, and synaptophysin was performed. The tumor showed partial positivity for INSM1 and chromogranin A, and positive staining for synaptophysin (**Fig. 5A**–**5F**), and about 40% positivity for MIB-1. Therefore, the gallbladder tumor was diagnosed as a metastatic lesion originating from the patient’s primary gastric cancer. The patient was discharged on postoperative day 9 without any complications. Upon his request, no postoperative chemotherapy was administered. However, 6 months later, multiple liver metastases developed, and the patient was started on chemotherapy with FOLFOX + Nivolumab regimen.

## DISCUSSION

Gallbladder metastasis is a relatively rare clinical occurrence. In one study analyzing 417 pathologically diagnosed malignant gallbladder tumors, 20 cases (4.8%) were identified as metastatic.^7)^ Previous reports have shown that the primary sites of gallbladder metastasis are diverse, including gastric cancer, colorectal cancer, pancreatic cancer, breast cancer, malignant melanoma, as well as renal cell carcinoma.^7–10)^ These variations in primary site distribution likely reflect regional differences in cancer incidence rates. To date, there have been six case reports of gallbladder metastasis from gastric cancer, including our case, reported in the English literature (**Table 1**).^4,11–14)^ Most of these cases were initially diagnosed as acute cholecystitis, whereas our case was suspected to be primary gallbladder cancer based on preoperative imaging.

**Table 1:** Clinical characteristics of patients with gallbladder metastasis of gastric cancer

No.	Age	Sex	Histological type of gastric cancer	Ly	V	N	Preoperative diagnosis	Surgery	Outcome	Reference
1	58	Male	sig	N/A	N/A	0	Acute cholecystitis	Cholecystectomy	11 months/Alive	^4)^
2	84	Male	well to mod	N/A	N/A	N/A	Acute cholecystitis	Subtotal cholecystectomy	N/A	^11)^
3	67	Male	sig	1	0	3	Gallbladder stone	Total gastrectomy and cholecystectomy	10 months/Alive	^12)^
4	72	Female	por	N/A	N/A	N/A	Acute cholecystitis	Total gastrectomy and cholecystectomy	N/A	^13)^
5	72	Male	por	N/A	N/A	N/A	Acute cholecystitis	Cholecystectomy	N/A	^14)^
6	81	Male	por	3	2	1	Gallbladder cancer	Extended cholecystectomy	15 months/Alive	Present case

Ly, lymphatic invasion; N, lymph node metastasis; N/A, not available; V, venous invasion

The diagnosis of gallbladder metastasis presents significant challenges. The CT imaging characteristics of gallbladder metastases typically mirror those of their primary tumors. Metastases from adenocarcinomas, including gastric cancer, characteristically display wall thickening with delayed contrast enhancement. By contrast, hypervascular tumors such as melanoma, hepatocellular carcinoma, and renal cell carcinoma typically demonstrate early wash-in and wash-out patterns.^3)^ The diagnostic challenge is further complicated by the fact that primary gallbladder tumors also exhibit wall thickening with delayed contrast enhancement, making CT findings of primary gallbladder tumors and adenocarcinoma metastases virtually indistinguishable.^3,5,6)^ Similarly, MRI characteristics show considerable overlap between primary gallbladder tumors and adenocarcinoma metastases.^3)^ Both typically appear iso-hypointense on T1-weighted images and slightly hyperintense on T2-weighted images.^3)^ In our case, the imaging findings lacked specific features diagnostic of gallbladder metastasis, highlighting the difficulty of preoperative diagnosis.

Histopathological and immunohistochemical evaluation is crucial for a definitive diagnosis of gallbladder metastasis. In our case, immunohistochemical staining revealed neuroendocrine marker expression, including INSM1, chromogranin A, and synaptophysin, in both the lymph node and gallbladder lesions. These markers were expressed by cells interspersed within the adenocarcinoma component. The adenocarcinoma component was morphologically dominant, while the neuroendocrine component was minimal. Thus, we diagnosed the tumor as gastric adenocarcinoma with neuroendocrine differentiation, a well-recognized histologic subtype characterized by focal neuroendocrine features.^15)^ Previous studies have reported that tumors with neuroendocrine differentiation were associated with higher rates of lymphovascular and perineural invasion, larger tumor size, and greater metastatic potential compared with conventional adenocarcinomas.^16)^ In our case, immunohistochemical staining for MUC5AC and MUC6 was negative in the primary gastric tumor, lymph node metastases, and gallbladder lesion, suggesting a common origin. Reduced expression of MUC5AC and MUC6 has been reported as a potential predictor of poor prognosis in gastric cancer.^17,18)^ Consistent with these evidence, the present case showed high malignant potential, with multiple recurrences.

The metastatic pathway from gastric cancer to the gallbladder remains complex and is not fully understood. Two case reports have proposed lymphatic spread as a likely mechanism. Lymph node metastasis and lymphovascular invasion can obstruct normal lymphatic flow, potentially leading to the formation of aberrant lymphatic channels that facilitate tumor spread to the gallbladder wall.^19,20)^ In our case, lymph node metastasis was identified around the cardia of the stomach and severe lymphatic invasion was observed at the specimen of ESD, supporting involvement of the lymphatic route. However, given the patient’s prior liver metastases, hematogenous spread cannot be excluded. A previous study on hepatocellular carcinoma reported that the majority of gallbladder metastases occurred via the portal venous system, suggesting hematogenous spread as the dominant route. It was speculated that tumor cells in the liver may migrate to the gallbladder through the portal venous circulation and form metastatic lesions within the gallbladder.^21)^ Although the primary tumor in the present case was gastric in origin, considering the previous history of liver metastasis, a similar hematogenous mechanism of metastasis via the portal venous system cannot be ruled out.

The management approach for gastric cancer with distant metastasis, particularly liver metastasis, has evolved from chemotherapy alone to a multidisciplinary treatment strategy incorporating liver surgery.^22–24)^ By contrast, there is no established consensus on the optimal treatment for gallbladder metastasis, primarily due to the limited number of reported cases. In the case of gallbladder metastasis derived from malignant melanoma, although selection bias needs to be considered, cholecystectomy can improve both prognosis and symptoms.^25)^ Another study also suggested that R0 resection may improve the survival outcomes.^7)^ In our case, extended cholecystectomy was performed based on a preoperative diagnosis of primary gallbladder cancer. However, when gallbladder metastasis is suspected preoperatively and no other metastatic sites are identified, simple cholecystectomy may be a reasonable alternative. While chemotherapy remains the standard treatment for metastatic gastric cancer, specific clinical circumstances may favor surgical intervention. Gallbladder metastasis presenting with acute cholecystitis has been associated with particularly poor outcomes. In cases where chemotherapy fails to control tumor progression and cholecystitis develops, surgical resection needs to be considered, as the combination of progressive disease and inflammatory complications may result in especially poor prognosis. Therefore, treatment decisions should be individualized, taking into account factors such as tumor location, size, and patient characteristics if the diagnosis of gallbladder metastasis was made before surgery.

## CONCLUSIONS

We herein reported a rare case of metachronous gallbladder metastasis from gastric cancer. Preoperative diagnosis of gallbladder metastasis is challenging due to its radiological similarity to primary gallbladder cancer.

## DECLARATIONS

### Funding

This work was supported by JSPS KAKENHI Grants (24K11920 to K.H. and 24K11898 to T.I.) and research grant from the Takeda Science Foundation (to K.H.). The funders had no role in study design, data collection and analysis, decision to publish, or preparation of the manuscript.

### Authors’ contributions

R.M. and K.H. acquired the data and drafted the manuscript.

M.Y., Y.S., M.T., S.O., M.M., K.F., and F.Y. were involved in drafting the manuscript.

T.I. critically revised the manuscript.

All authors read and approved the final manuscript.

### Availability of data and material

All data generated or analyzed during this study are included in this published article.

### Ethics approval and consent to participate

This work does not require ethical considerations or approval. Informed consent to participate in this study was obtained from the patient.

### Consent for publication

The patient has given consent for the publication of images.

### Competing interests

The authors declare that they have no competing interests.
